# Discovery of ERα‐Targeting Phytochemicals With In Vitro Cytotoxicity and Computational Prediction of Y537S Mutant Inhibition

**DOI:** 10.1111/cbdd.70393

**Published:** 2026-09-03

**Authors:** Dejun Jiang, Oh Wook Kwon, Hanbin Joe, Euijeong Shin, Sungjoon Cho, Hyuk‐Ku Kwon, Youngjin Choi

**Affiliations:** ^1^ Department of Environmental Engineering Hoseo University Asan Republic of Korea; ^2^ Pet‐Loss Center Hoseo University Asan Republic of Korea; ^3^ Department of Food Science & Technology Hoseo University Asan Republic of Korea; ^4^ Department of Bio‐Applied Toxicology Hoseo University Asan Republic of Korea

**Keywords:** breast cancer, cytotoxicity, estrogen receptor alpha, molecular dynamics simulations, phytochemicals, Y537S mutation

## Abstract

Breast cancer is known as a frequently diagnosed malignancy in women. Over 70% of cases express estrogen receptor α (ERα). The activation of ERα promotes tumor proliferation and progression. Furthermore, mutations in ERα lead to acquired resistance against standard endocrine therapies such as tamoxifen. The induced resistance posed a significant clinical challenge in metastatic breast cancer. In this study, research for identifying novel, naturally derived compounds to inhibit the Y537S‐mutated ERα was conducted using in silico methods supported by an in vitro cytotoxicity screen. Molecular docking and molecular dynamics simulations served as the primary in silico screening strategies. The top 2% of candidates were filtered from a docking screen of the IBS natural library composed of over 15,000 chemicals. From this group, 11 compounds were purchased and tested using a cell‐based cytotoxicity assay in MCF‐7 before advancing to detailed simulations. Five candidates were then advanced to 150 ns MD simulations. A post‐MD analysis followed, including MM‐PBSA binding free energy calculations and principal component analysis (PCA). The phytochemical ibs‐04156 was identified as the most promising overall candidate, predicted to maintain consistent stability across both the wild‐type and Y537S‐mutated ERα, while ibs‐18821 demonstrated potent mutant‐specific inhibition via an allosteric mechanism involving spatial deviations in distal helices H3 and H11. This result shows that mutated ERα can be potentially targeted by bioactive phytochemical scaffolds found through molecular modeling, presenting a potential therapeutic strategy for metastatic breast cancer resistant to therapies such as tamoxifen.

## Graphical abstract

Integrating high‐throughput virtual screening, cytotoxicity assays, and molecular dynamics simulations, this study identifies the phytochemical ibs‐04156 as a reliable dual‐action candidate across both wild‐type and mutated estrogen receptor alpha (ERα), while ibs‐18821 emerges as a potent allosteric inhibitor specifically targeting the drug‐resistant Y537S mutant.

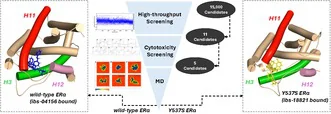

## 1 Introduction

Breast cancer is considered the most frequently diagnosed malignancy in women today (Arnold et al. 2022; Bray et al. 2024; Siegel et al. 2025). This type of cancer is highly associated with germline variants, including BRCA1, BRCA2, and estrogen‐related signaling pathways (Ferreyra et al. 2023). Over 70% of breast cancer tumors express a ligand‐activated hormone receptor, estrogen receptor α (ERα) (Porras et al. 2021). Its activation causes cellular proliferation and tumor progression in hormone‐receptor‐positive breast cancer (Liu et al. 2020). Hormone therapy is a standard treatment for ERα‐positive breast cancer by blocking the estrogen receptor with selective estrogen receptor modulators (SERM) and degraders (SERD) (Kim and Lukong 2025; Lumachi et al. 2013). Tamoxifen, a widely used SERM, functions as an ERα inhibitor in breast tissue, thereby contributing to the hormone therapy (Loi et al. 2010; Wu et al. 2018). This treatment is effective and has a lower recurrence rate (Fisher et al. 1996; Lazzeroni et al. 2023). However, a major clinical challenge is acquired resistance, as most metastatic cancers stop responding to the therapy while continuing to express ERα (Chang 2012; Fan and Jordan 2019).

Acquired endocrine resistance is proven to be caused by mutations in the ESR1 gene, which encodes ERα (Ahn et al. 2022; Jeselsohn et al. 2015). These mutations are a key resistance mechanism and are found in 20%–40% of patients with ER+ metastatic breast cancer after progression on hormone therapy (Palaniappan 2024). ESR1 mutations are rare in primary, untreated tumors (Kinslow et al. 2022). This indicates that the selective pressure of hormone therapy drives the acquisition of cancer cells with these mutations (Merenbakh‐Lamin et al. 2013). Most of these ESR1 mutations occur within the ERα ligand‐binding domain (Toy et al. 2013). A well‐defined hotspot exists at residues L536, Y537, and D538 (Harrod et al. 2022). Among these residues, Y537S is one of the most prevalent point mutations (Dustin et al. 2019). Previous studies demonstrate that these mutations stabilize the receptor in an active agonist conformation in the absence of estrogen (Toy et al. 2013). This provides a molecular explanation for the mutants' constant activity and their attenuated response to tamoxifen (Harrod et al. 2022).

New research aims to identify novel antagonists that can inhibit these mutated forms of ERα. Some have focused on naturally occurring chemicals that exhibit endocrine activity, as well as a proven safety profile (Del Pup et al. 2016; Eve et al. 2020). Various food‐derived substances have been found to modulate estrogen receptors. Muthyala et al. (2004) demonstrated that equol, a bacterial metabolite of the soybean isoflavone, exhibits selective affinity for ERα and Erβ. Subsequent investigations cataloged additional food‐derived chemicals—including curcumin, genistein, apigenin, resveratrol, ellagic acid, sulforaphane, quercetin, and biochanin A—that modulate estrogen receptor (Bak et al. 2016; Kunnumakkara et al. 2008; Lecomte et al. 2017; Nwachukwu et al. 2014). However, no specific phytochemical has been validated as a potent antagonist against mutated ERα.

Using a combination of molecular docking and dynamics simulations to screen a commercial compound library has been proven to be a reliable strategy in exploring potential protein inhibitors (Alanzi et al. 2025; Da Fonseca et al. 2024). As a computer‐aided drug discovery (CADD) strategy, this combination offers various advantages, which include cost‐efficient calculations, a lower false‐positive rate, and support for various protein targets (Liu et al. 2025; Wei and McCammon 2024). The docking study provides primary affinity scores for later dynamics, as well as the initial starting coordinates in the binding pocket (Chen et al. 2014). The MD study verifies the potency of the hit drugs and provides dynamic insights into drug–protein complexes through post‐MD analysis (Durrant and McCammon 2011). The binding free energy calculated via MM‐PBSA provides an overall binding affinity for each hit chemical, offering a possibility as inhibitors (Wang et al. 2019).

In this study, over 15,000 phytochemicals were initially screened via molecular docking. Lead candidates were then experimentally evaluated for in vitro cytotoxicity using the ERα‐positive MCF‐7 cell line, which expresses wild‐type ERα. To predict their ability to overcome endocrine resistance, the most active compounds were advanced to 150 ns molecular dynamics (MD) simulations. These simulations characterized their binding profiles against both the wild‐type and Y537S‐mutated receptors, computationally assessing their potential as mutant‐specific inhibitors.

## 2 Materials and Methods

### 2.1 Structural Resources

In this study, the 3D structure of wild‐type estrogen receptor alpha (PDB ID: 3ERT) and its Y537S mutant (PDB ID: 7UJ8) were obtained from the RCSB Protein Data Bank. These structures were resolved via X‐ray by Shiau et al. (1998) and by Hosfield et al. (2022), respectively. The screening library containing 15,431 phytochemicals was sourced from the InterBioScreen (IBS) phytochemical database (Rampogu et al. 2021). The 3D structures of the reference ligands, 4‐hydroxytamoxifen and lasofoxifene, were obtained from the PubChem database. The receptor's 3D structure was pre‐cleaned by removing water molecules and nonprotein heavy atoms using PyMOL 3.1.3 (Wang et al. 2025). The missing atoms in the studied region were fixed by UCSF Chimera 1.18 (Pettersen et al. 2004). Kollman partial charges were assigned to the protein structure and converted to PDBQT format for subsequent docking studies with AutoDock (Theodore et al. 2023). In terms of IBS library drugs, hydrogen atoms were added using Open Babel before converting the structures to PDBQT format (O'Boyle et al. 2011).

### 2.2 Molecular Docking

A high‐throughput molecular docking study on 15,431 natural compounds was conducted by GPU‐accelerated AutoDock4 (Morris et al. 2009). The grid box for general ERα was placed at coordinates of 30.966, −1.528, and 24.626 Å for the *X*, *Y*, and *Z* axes with a cubic search area of 30 Å for each edge. The lowest docking affinity served as the representative binding score. The binding pocket was validated using web‐based PrankWeb and CASTp. The search box size was further evaluated with eBoxSize (Binkowski 2003; Jendele et al. 2019).

### 2.3 Cells and Reagents

MCF‐7 human breast cancer cells (KCLB, Republic of Korea) were cultured in RPMI‐1640 medium (Gibco BRL, USA) at 37°C with 5% CO_2_ (Razak et al. 2019). The culture medium consisted of 10% fetal bovine serum (FBS) and 100 U/mL penicillin–streptomycin (Gibco BRL, USA) (Liu et al. 2023). The 3‐(4,5‐dimethylthiazol‐2‐yl)‐2,5‐diphenyltetrazolium bromide (MTT) reagent (Sigma‐Aldrich, USA) was primarily used during the cytotoxicity assay in this study (Ghasemi et al. 2021).

### 2.4 Cytotoxicity Screening

The MTT assay was used to assess the cytotoxicity of the leading candidate compounds identified from the high‐throughput docking study (Sarı 2021). Breast cancer cells were seeded with a density of 1 × 10^4^ cells per well, consisting of 100 μL of growth medium, and exposed to each candidate compound for 48 h (Cabrera‐Licona et al. 2024). Afterward, 5 mg/mL MTT solution was added and incubated at 37°C for an additional 4 h (Omar et al. 2011). Formazan crystals produced by viable cancer cells were solubilized, and their absorbance was measured at 570 nm (Benov 2021).

### 2.5 Molecular Dynamics Simulation

Molecular dynamics (MD) simulations were slightly modified from the protocol designed by Jiang et al. (2025). Four‐stage simulations were performed in AMBER24 sequentially, including energy minimization, thermal‐NVT equilibration, mini‐NPT equilibration, and production (Case et al. 2023). Initially, the system underwent 5000 cycles of energy minimization to eliminate steric clashes. The temperature was raised from 100 to 310 K gradually during the heating phase. An additional 1 ns of short NPT equilibration ensured the stability of pressure and density before the major production run. In the end, a 150 ns production trajectory containing 15,000 frames was created to analyze the complex's long‐term dynamical behavior. For the Y537S mutant systems, lasofoxifene was also subjected to this MD protocol to serve as a clinically relevant, mutant‐active reference control. The complete protocol was repeated three times for key candidates to minimize statistical uncertainty.

### 2.6 Free Binding Energy Calculations

The molecular mechanics Poisson–Boltzmann surface area method in AMBER was used for binding free energy calculations (Miller et al. 2012). The binding free energy (Δ*G*_TOTAL_) was determined by a combination of the gas phase interaction term and the solvation correction (Case et al. 2023). The gas phase contribution was calculated using Equation (1):

(1) $$∆G_{\text{vacuum}}=E_{\mathrm{MM}}$$

where *E*_MM_ signifies the molecular mechanics energy. The entropic contribution was not included in these calculations; this omission is acknowledged as a limitation of the current approach, though it is often excluded in comparative MM‐PBSA studies to prevent high fluctuations from disrupting the relative predictive rankings of the ligands.

The solvation term was determined using Equation (2):

(2) $$∆G_{\text{solv}}=G_{\text{electro}\_80}-G_{\text{electro}\_1}+{\Delta G}_{\text{hydrophobic}}$$

where *G*_electro_80_ represents the electrostatic solvation free energy at a dielectric constant of 80, *G*_electro_1_ denotes the electrostatic energy in vacuum with the dielectric constant of 1. Δ*G*_hydrophobic_ signifies the nonpolar contribution from hydrophobic effects. The overall binding free energy was calculated by integrating the gas phase and solvation correction, as presented in Equation (3):

(3) $${\Delta G}_{\text{Total}}={\Delta G}_{\text{vacuum}}+{\Delta G}_{\text{solv}}$$

### 2.7 Principal Component Analysis Methodology

The principal component analysis (PCA) was conducted using the AMBER cpptraj module (Case et al. 2023). The production trajectory was aligned to the initial frame using the Cα atoms of all 247 residues to remove global rotational and translational motion. A covariance matrix was constructed from the atomic fluctuations of these Cα atoms. This matrix was subsequently diagonalized to yield corresponding eigenvalues and eigenvectors. The first two eigenvectors, PC1 and PC2, representing the largest collective motions in the system, were extracted as the principal components. The simulation trajectory and the binding energy of each frame were projected onto these two principal components to visualize the dominant motions along each vector.

## 3 Results

### 3.1 High‐Throughput Screening

**FIGURE 1:**
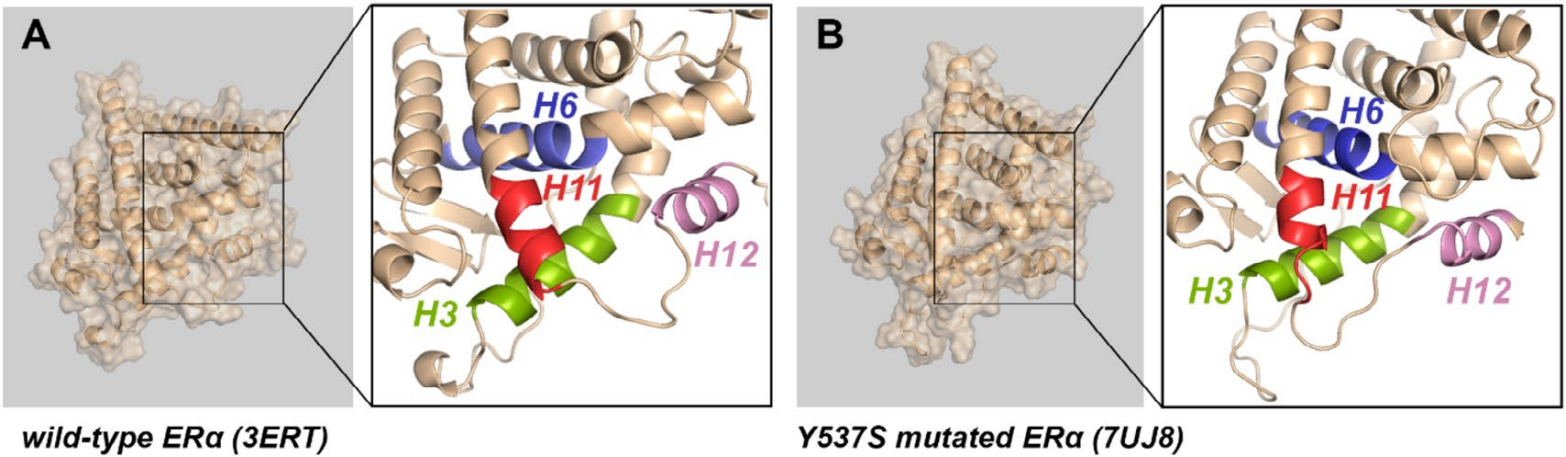
The crystal structure of the wild‐type (PDB ID: 3ERT) and Y537S mutated (PDB ID: 7UJ8) estrogen receptor alpha. The inset highlights the key alpha‐helices—H3, H6, H11, and H12—that comprise the ligand‐binding region.

In this study, high‐throughput virtual screening was performed using AutoDock4 against the estrogen receptor α by docking the entire IBS phytochemical database. The docking study provides an efficient primary filter to identify compounds capable of binding to the general ERα ligand pocket. The natural library comprises 15,431 naturally derived compounds. The metabolite of tamoxifen, 4‐hydroxytamoxifen, served as the reference ligand, and Figure 1 illustrates the binding pocket of the established wild‐type and Y537S‐mutated ERα.

**FIGURE 2:**
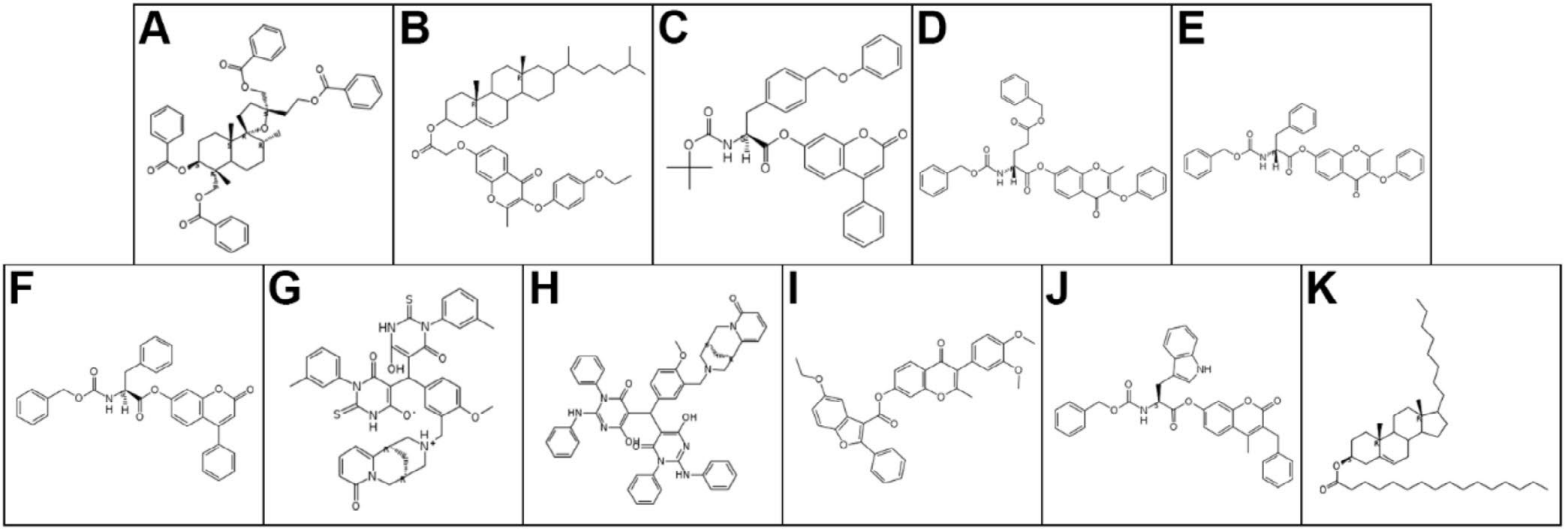
Chemical structures of the key phytochemical candidates derived from high‐throughput screening for ERα inhibition: (A) ibs‐00055, (B) ibs‐00791, (C) ibs‐04156, (D) ibs‐07025, (E) ibs‐07124, (F) ibs‐08925, (G) ibs‐10717, (H) ibs‐18821, (I) ibs‐19591, (J) ibs‐31168, and (K) ibs‐37312.

The comprehensive docking study yielded the affinity distribution shown in Figure S1. Most ligands produced binding energies ranging from −6.0 to −10.0 kcal/mol, resulting in a mean value of −8.30 ± 2.03 kcal/mol. The strongest interaction was predicted for ibs‐00791 at −15.05 kcal/mol, while a small number of compounds exhibited unfavorable scores higher than −3.00 kcal/mol. A practical threshold of −12.00 kcal/mol was set for subsequent selection, retaining 253 molecules. These selected molecules account for approximately 2% of the screened library. The potential candidate was visually examined in PyMol 3.1.3 and evaluated for drug likeness and commercial availability (Wang et al. 2025). Eleven ligands met the criteria and were advanced for further study, as shown in Figure 2: ibs‐00055, ibs‐00791, ibs‐04156, ibs‐07025, ibs‐07124, ibs‐08925, ibs‐10717, ibs‐18821, ibs‐19591, ibs‐31168, and ibs‐37312. Their calculated binding affinities were −14.98, −15.05, −12.61, −12.89, −12.56, −12.05, −12.29, −13.61, −14.30, −12.57, and −13.69 kcal/mol, respectively. The properties of these compounds, including their chemical class and origin, are summarized in Table 1.

**TABLE 1:** Physicochemical properties and docking affinities of selected hit compounds.

Chemical ID	Chemical class	Origin	M.W. (g/mol)	Affinity (kcal/mol)
ibs‐00055	Spiro‐diterpenoid benzoate	Fungi	772.9	−14.98
ibs‐00791	Sterol‐flavonoid ester	Animal cells	753.0	−15.05
ibs‐04156	Neoflavonoid‐amino acid ester	Rosewoods	591.6	−12.61
ibs‐07025	Flavone‐glutamic acid ester	Parsley	621.6	−12.89
ibs‐07124	Sterol palmitate ester	Plant oils	625.1	−12.56
ibs‐08925	Flavone‐phenylalanine ester	Parsley	549.6	−12.05
ibs‐10717	Neoflavonoid‐phenylalanine ester	Rosewoods	519.5	−12.29
ibs‐18821	Tetrahydropyrimidine alkaloid	Lupins	788.9	−13.61
ibs‐19591	Bispyrimidine alkaloid	Lupins	879.0	−14.30
ibs‐31168	Isoflavone‐benzofuran ester	Soybean	576.6	−12.57
ibs‐37312	Coumarin‐tryptophan ester	Tonka bean	586.6	−13.69

### 3.2 Cytotoxicity Screening Results

**FIGURE 3:**
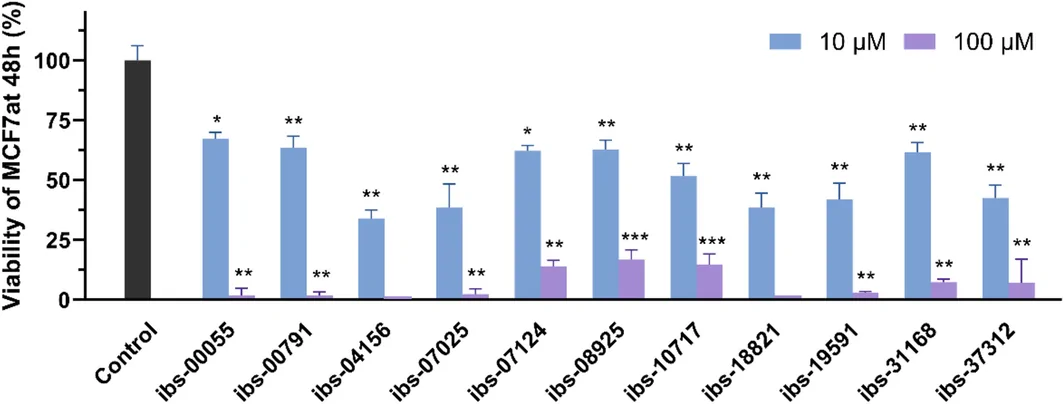
Dose‐dependent cytotoxicity of hit compounds on MCF‐7 cells at 10 and 100 μM concentrations. Mean percentage viability (±SEM; *n* = 3) after 48‐h treatment relative to untreated control. Significance determined by Welch's *t*‐test (*p < 0.05, **p < 0.01, ***p < 0.001).

The cytotoxic potential of 11 natural compounds from the IBS library was evaluated in the ERα + MCF‐7 cell line to determine their biological activity. As shown in Figure 3, MCF‐7 ERα + breast cancer cells were exposed to each compound at concentrations of 10 and 100 μM for 48 h. The absorbance was measured spectrophotometrically as follows. At 10 μM, the mean cell viability across all candidates was 51.33% ± 12.45%, with five compounds—ibs‐04156, ibs‐07025, ibs‐18821, ibs‐19591, and ibs‐37312—reducing viability to below 50%. Increasing the concentration to 100 μM reduced the mean viability to 6.25% ± 6.23%, while four compounds—ibs‐00055, ibs‐00791, ibs‐04156, and ibs‐18821—decreased viability to below 2%. Based on the consistent cytotoxicity observed in wild‐type ERα‐expressing MCF‐7 cells, five compounds—ibs‐04156, ibs‐07025, ibs‐18821, ibs‐19591, and ibs‐37312—were selected for subsequent molecular dynamics simulations. These simulations were conducted to investigate their kinetic interactions with both the wild‐type receptor and the Y537S mutant, computationally predicting their potential to overcome acquired endocrine resistance.

### 3.3 Post‐MD Analysis

#### 3.3.1 RMSD, RMSF, and *R*_g_

**FIGURE 4:**
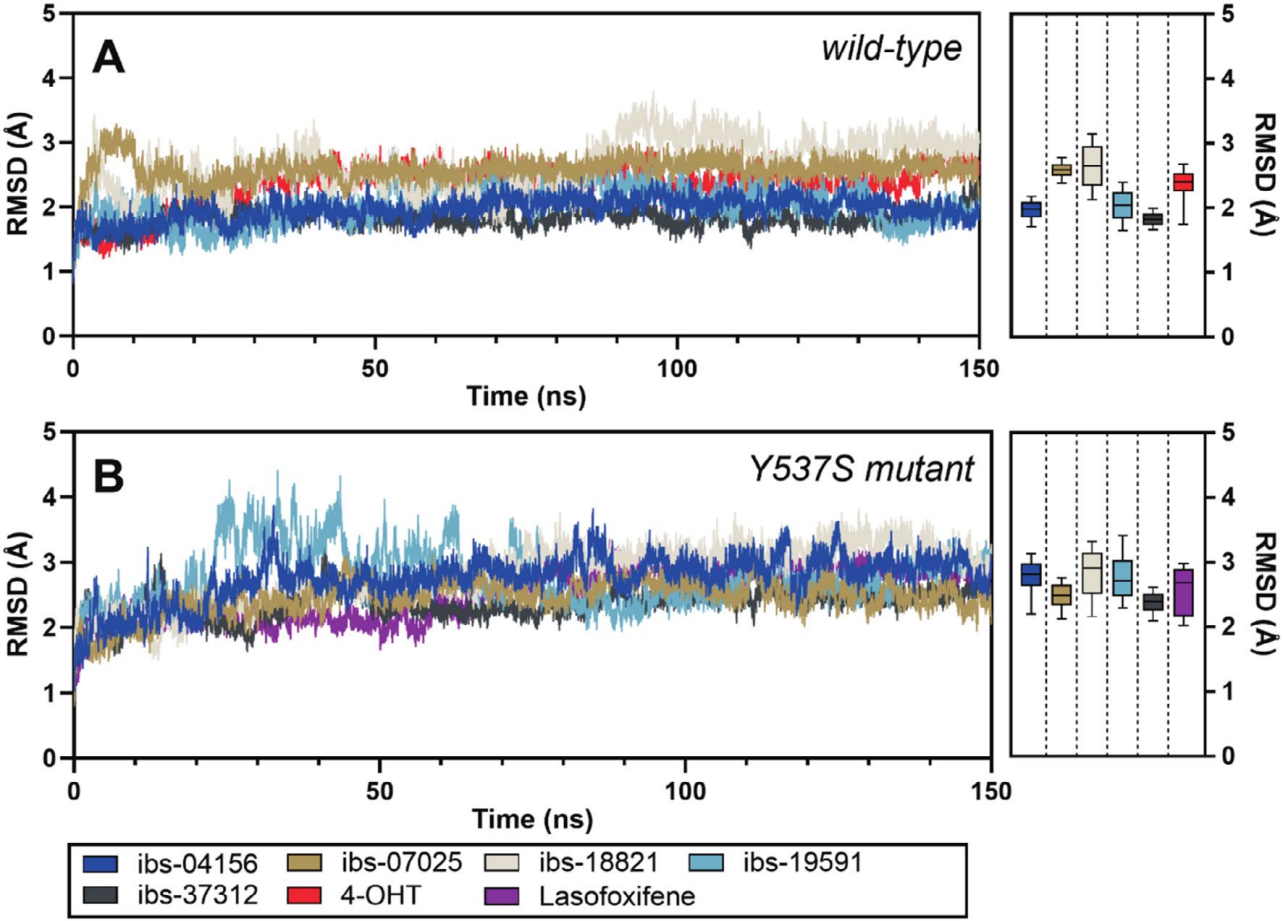
RMSD of wild‐type ERα (A) and its Y537S mutant (B) complex with five leading chemicals and clinical controls (4‐OHT and lasofoxifene) during 150 ns MD simulations, along with the box and whiskers (10%–90%) plot for the last 50 ns.

Root‐mean‐square deviation (RMSD) was calculated for 12 distinct systems. As illustrated in Figure 4, these systems are constituted by the complexation of six principal compounds, along with their respective reference chemicals (4‐hydroxytamoxifen and lasofoxifene), with both the wild‐type and mutant forms of the estrogen receptor alpha protein. In the wild‐type complex, compounds ibs‐37312 and ibs‐04156 exhibited the lowest root‐mean‐square deviation values of 1.83 ± 0.14 and 2.02 ± 0.13, respectively. These results are indicative of a highly stable binding conformation. The stability of these compounds exceeded that of the reference chemical, 4‐OHT, which presented an RMSD of 2.46 ± 0.17. In contrast, ibs‐18821 and ibs‐07025 demonstrated comparatively lower stability, with corresponding RMSD values of 2.95 ± 0.19 and 2.62 ± 0.12. Conversely, a different profile of molecular stability was observed in the Y537S mutant system. Within this mutated complex, lasofoxifene registered an RMSD value of 2.90 ± 0.10. It was exceeded in stability by ibs‐37312 and ibs‐07025, which yielded lower RMSD values of 2.50 ± 0.11 and 2.52 ± 0.18, respectively. These latter values suggest the formation of more stable binding complexes relative to the clinical control in mutated systems.

**FIGURE 5:**
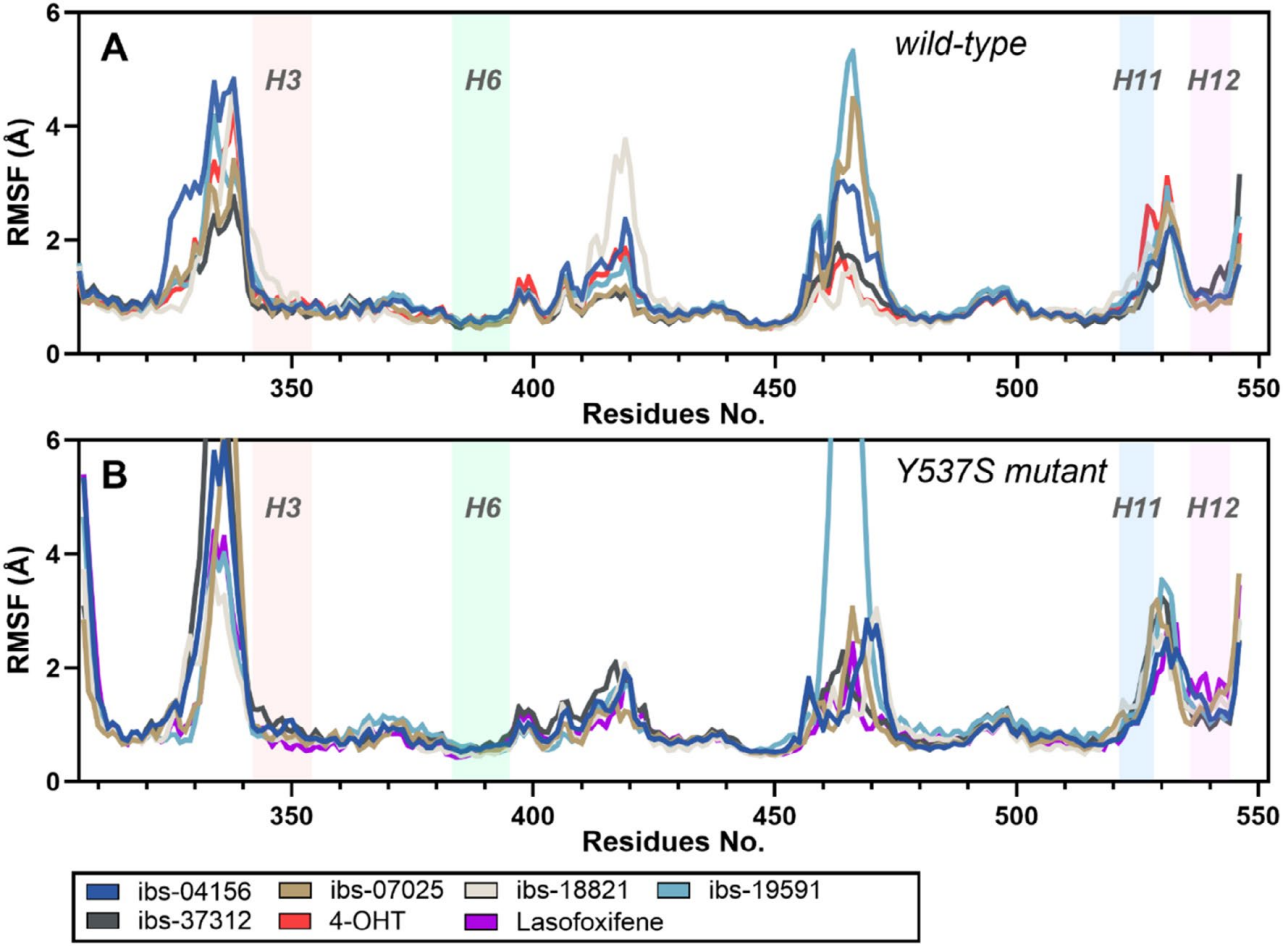
RMSF of the wild‐type (A) and mutant (B) estrogen receptor alpha complexed with the phytochemical candidates and clinical controls during 150 ns molecular dynamics simulations. The key alpha‐helices, H3, H6, H11, and H12, are highlighted.

The root‐mean‐square fluctuation (RMSF) serves as a key indicator of residue flexibility upon complexation with ligands. The binding region of estrogen receptor alpha is constituted by four alpha‐helices, namely H3, H6, H11, and H12, as illustrated in Figure 5. The residue ranges for these helices are as follows: H3 comprises residues 342–354, H6 encompasses residues 383–395, H11 includes residues 521–528, and H12 contains residues 536–544. Among these, H12 is considered the gate of the ERα binding pocket, and its flexibility directly influences the efficacy of ligand binding. The Y537S mutation is located at residue 537 within H12. A significant increase in the flexibility of this region was observed post‐mutation. In complexes with ibs‐07025 and ibs‐18821, the average RMSF of the H12 region increased from the wild‐type values of 0.89 ± 0.06 and 0.94 ± 0.07 Å to 1.37 ± 0.23 and 1.33 ± 0.09 Å, respectively. Conversely, a decrease was noted for ibs‐37312, from 1.24 ± 0.22 to 1.09 ± 0.09 Å. Variations in flexibility were also observed in other alpha‐helical regions. The average RMSF of the H3 region exhibited a general downward trend. Specifically, the RMSF for the ibs‐18821 complex decreased from 1.22 ± 0.42 to 0.79 ± 0.10 Å. In contrast, the RMSF for the ibs‐37312 complex increased from 0.76 ± 0.07 to 1.02 ± 0.18 Å. Other systems showed minimal changes. The H11 alpha‐helix, adjacent to the H12 region, demonstrated an overall increase in its average RMSF. For the ibs‐37312 and ibs‐07025 complexes, the RMSF values rose from 0.89 ± 0.21 and 1.03 ± 0.37 Å to 1.57 ± 0.68 and 1.53 ± 0.71 Å, respectively. The flexibility of residues within the H6 region was not significantly impacted by the mutation. Significant alterations in residue flexibility were also observed in regions outside of the key alpha‐helices. Notably, within the residue segment 462–468, the RMSF value for the mutant complex involving ibs‐19591 reached a high value of 10.64 Å. Conversely, a reduction in residue flexibility was recorded in the region spanning residues 416–423. For the wild‐type receptor complexed with ibs‐18821 and ibs‐04156, the RMSF values decreased from 3.76 and 2.36 Å to 2.06 and 1.93 Å in the mutant, respectively.

**FIGURE 6:**
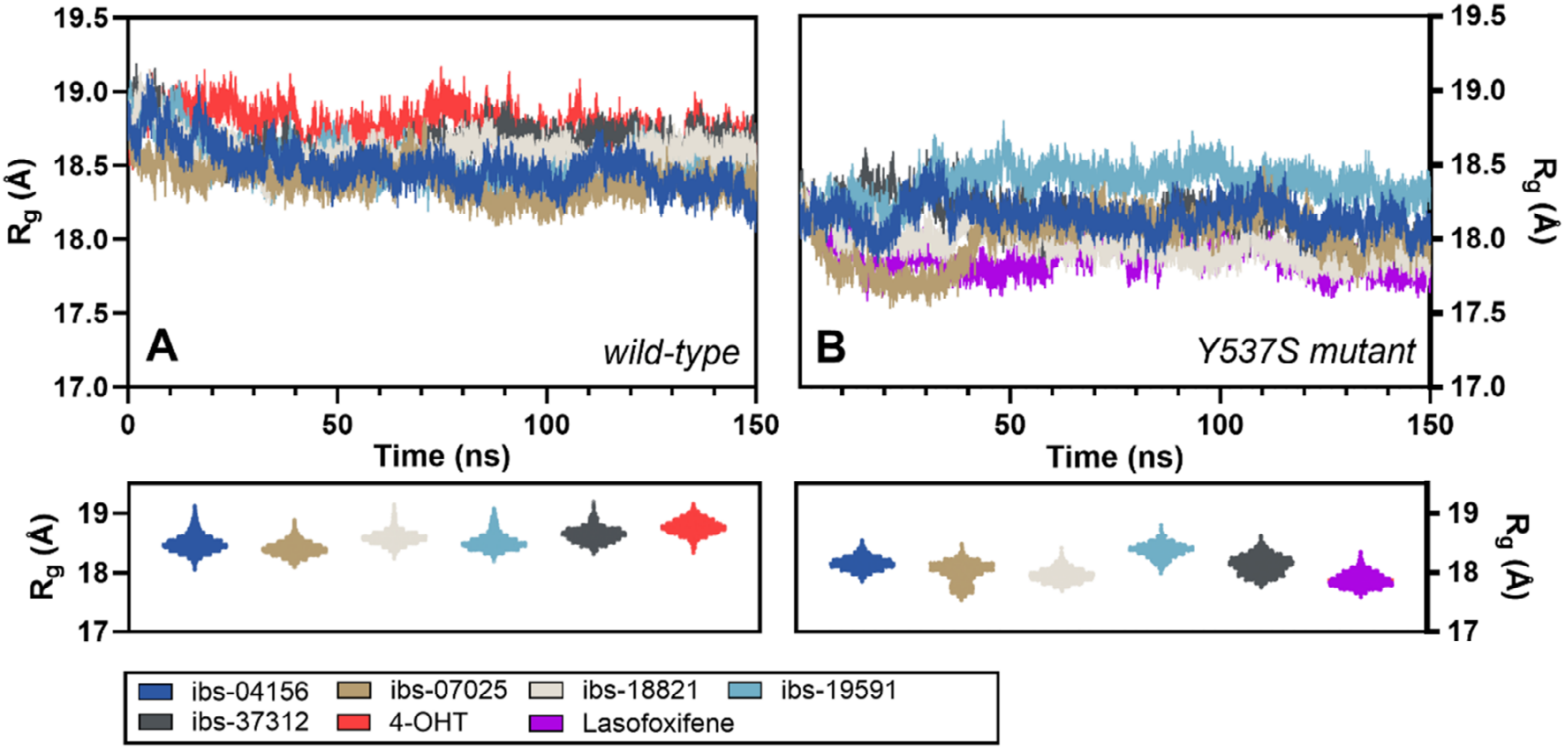
Comparative analysis of the *R*_g_ for the protein backbone of (A) wild‐type estrogen receptor alpha and (B) the Y537S mutant ERα. The analysis was conducted on systems complexed with the phytochemical candidates and clinical controls over a 150 ns simulation period; the upper panels show the time evolution of the *R*_g_ values, while the lower panels display the corresponding probability distribution for each system.

The radius of gyration (*R*_g_) was analyzed to assess the degree of unfolding or compaction of the overall structure of the ERα in complex with various chemical compounds, both prior to and following a specific mutation. In the wild‐type complex systems, ibs‐07025 and ibs‐04156 exhibited comparatively low mean *R*_g_ values of 18.35 ± 0.07 and 18.40 ± 0.11 Å, respectively, as shown in Figure 6. In contrast, the *R*_g_ of the reference chemical, 4‐hydroxytamoxifen, was 18.70 ± 0.11 Å, which represented the highest *R*_g_ value among the six wild‐type complexes analyzed. Subsequent to the introduction of the Y537S mutation, the conformations of these complexes underwent varying degrees of alteration. A general trend of compaction was observed across all systems, including the one complexed with the mutant‐active control lasofoxifene. Notably, the *R*_g_ of the complex containing ibs‐18821 decreased substantially from 18.58 ± 0.07 to 17.88 ± 0.07 Å, rendering it a highly compact system comparable to lasofoxifene. Following this, the complexes with ibs‐37312 and ibs‐07025 also demonstrated significant reductions in their radius of gyration values, which decreased to 18.01 ± 0.11 and 18.05 ± 0.13 Å, respectively. These results collectively indicate that the Y537S mutation induces a pronounced conformational compaction in the ERα–ligand complexes.

#### 3.3.2 H‐Bonds and Contacts

**FIGURE 7:**
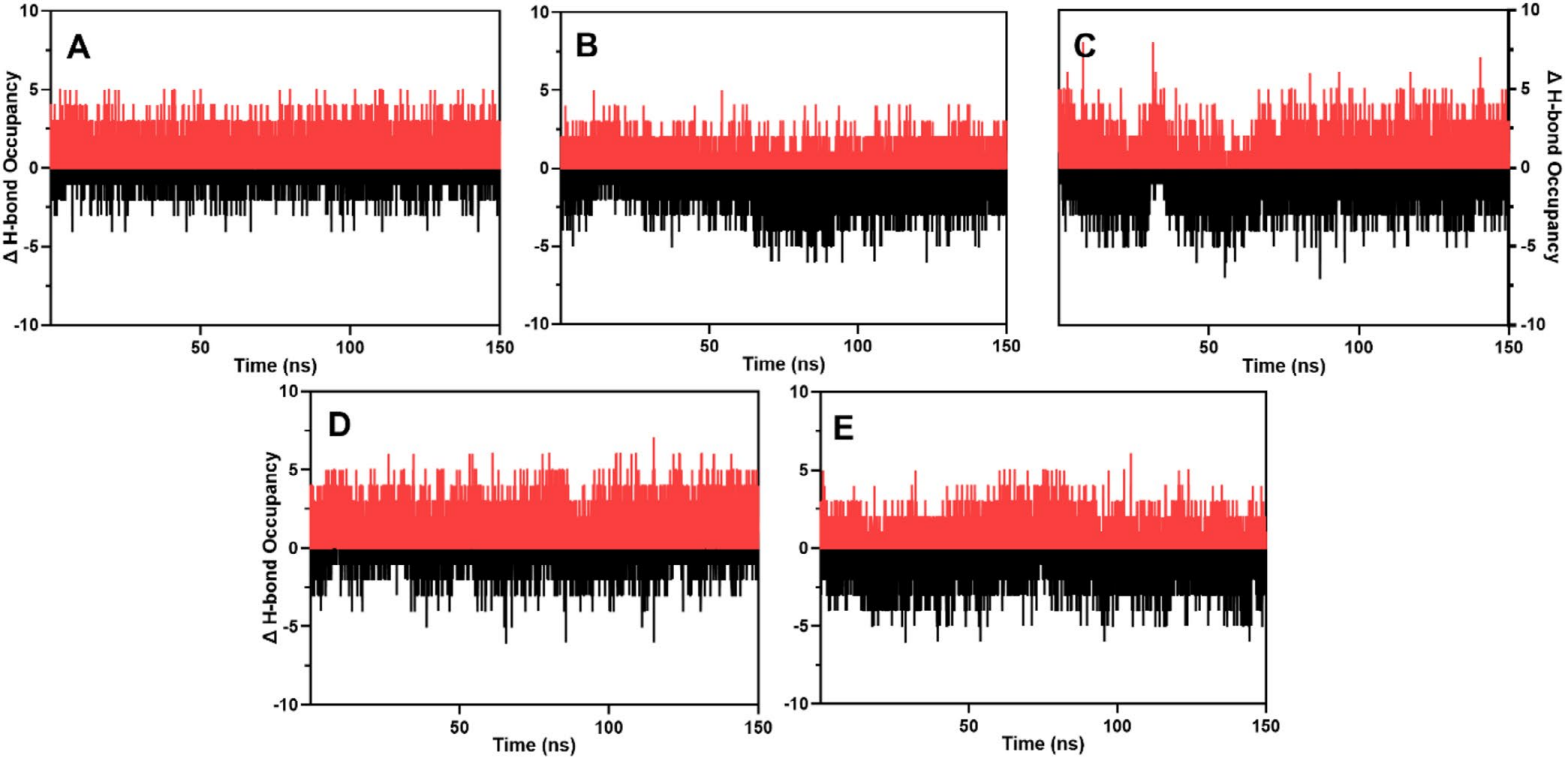
Differential hydrogen bond occupancy between the Y537S mutant and the wild‐type ERα when complexed with selected hit candidates, monitored over a 150 ns molecular dynamics simulation. The specific ligand‐receptor systems are as follows: (A) ibs‐04156, (B) ibs‐07025, (C) ibs‐18821, (D) ibs‐19591, and (E) ibs‐37312; positive values (red) indicate a greater number of hydrogen bonds in the mutant system compared to the wild‐type; conversely, negative values (black) denote a lower number of hydrogen bonds in the mutant system.

The number of hydrogen bonds between estrogen receptor α and its ligands provides critical insights into the binding energy and affinity. In the present study, the number of H‐bonds was calculated for both the wild‐type and mutant systems. Figure 7 illustrates the difference in the number of H‐bonds between the mutant and wild‐type systems. A positive value, indicated in red, signifies a greater number of H‐bonds in the mutant system, whereas a negative value, indicated in black, denotes a lesser number.

An analysis of the five systems reveals that most systems exhibited an increase in H‐bond numbers following mutation, while ibs‐37312 showed a decrease. Specifically, the most significant increase was observed for ibs‐19591, where the mean number of H‐bonds rose from 0.71 ± 0.94 to 1.95 ± 1.03. A notable increase was also recorded for ibs‐04156, with the mean H‐bond number changing from 0.44 ± 0.72 to 1.13 ± 0.96. In contrast, the mean H‐bond numbers for ibs‐07025 and ibs‐18821 remained relatively stable post‐mutation, at 0.49 ± 0.77 and 2.15 ± 1.26, respectively, indicating minimal difference between the wild‐type and mutant states.

Conversely, a reduction was noted for ibs‐37312, with the average number of H‐bonds decreasing from 1.46 ± 1.11 to 0.99 ± 0.92.

**FIGURE 8:**
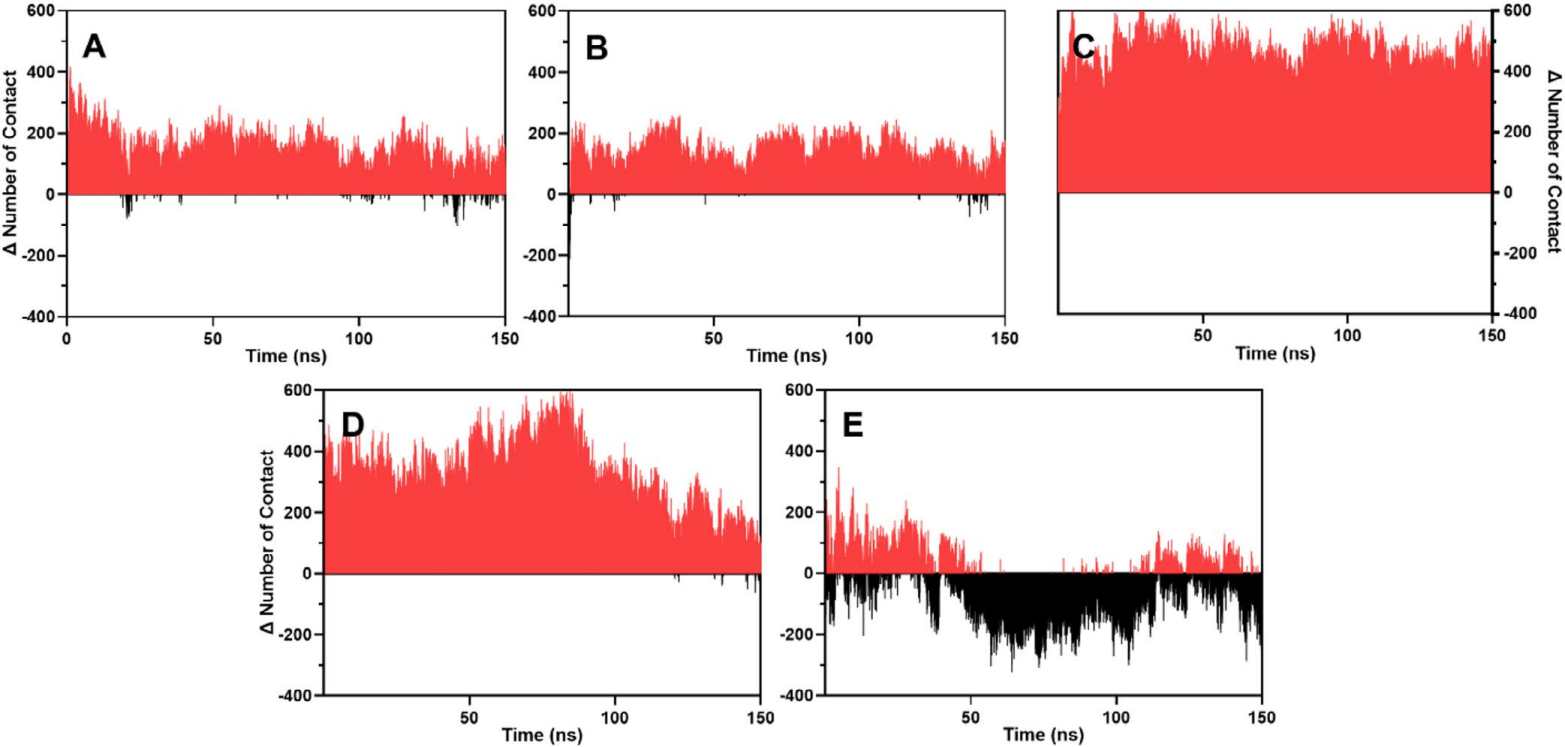
The difference in the number of atomic contacts between the wild‐type and mutant ERα, complexed with selected lead compounds. The data were recorded over a 150 ns molecular dynamics simulation for the following systems: (A) ibs‐04156, (B) ibs‐07025, (C) ibs‐18821, (D) ibs‐19591, and (E) ibs‐37312; positive values (red) represent a greater number of contacts in the mutant system relative to the wild‐type; conversely, negative values (black) represent a smaller number of contacts in the mutant system.

Contact frequency serves as a metric for the overall proximity between the estrogen receptor alpha protein and its ligands. In this investigation, a contact was defined as any atom pair between the protein and the ligand with a separation distance of < 4.5 Å. As illustrated in Figure 8, the difference in the number of contacts was calculated to provide a direct comparison of the protein–ligand interactions before and after the Y537S mutation over the course of a 150 ns molecular dynamics simulation.

The analysis reveals that for the majority of the systems, the contact frequency increased significantly following the mutation; the ibs‐37312 system was a notable exception. The most substantial increase was observed for ibs‐18821, for which the mean number of contact pairs rose from 105.67 ± 25.07 to 514.37 ± 46.95. A significant increase was also recorded for ibs‐19591, from 267.47 ± 39.28 pairs to 430.62 ± 57.87 pairs. The mean contact pairs for the remaining systems, ibs‐04156 and ibs‐07025, increased to 293.00 ± 35.55 and 259.76 ± 36.42, respectively. Conversely, the ibs‐37312 system demonstrated a decrease in interaction, with the average number of contact pairs declining from 291.44 ± 63.31 to 252.43 ± 32.28.

#### 3.3.3 Binding Free Energy Analysis

The binding free energies of the ERα–ligand complexes were calculated using the Molecular Mechanics Poisson–Boltzmann Surface Area method on trajectories generated with AMBER24. This calculation involves summing the vacuum interaction enthalpy, Δ*G*_gas_, and the solvation free energy, Δ*G*_solv_, to determine the total binding free energy, ΔTotal.

The calculated binding affinities for the ligands with wild‐type ERα are presented in Table 2. For the wild‐type receptor, 4‐OHT demonstrated the highest binding affinity, with a ΔTotal of −9.93 ± 4.79 kcal/mol. Following 4‐OHT, ibs‐04156 and ibs‐37312 yielded moderate binding energies of −4.61 ± 5.73 kcal/mol and −3.59 ± 4.94 kcal/mol, respectively. Notably, the interactions for ibs‐18821 and ibs‐19591 became unfavorable, as indicated by their positive ΔTotal values of 3.53 ± 4.74 and 3.65 ± 5.43 kcal/mol. This suggests a loss of binding affinity for these specific ligands to the wild‐type ERα.

**TABLE 2:** Binding free energy components (kcal/mol) for wild‐type ERα–ligand complexes calculated via the MM‐PBSA method.

Component	ibs‐04156	ibs‐07025	ibs‐18821	ibs‐19591	ibs‐37312	4‐OHT
VDW	−68.44 ± 3.56	−72.81 ± 3.25	−40.04 ± 4.86	−74.77 ± 4.32	−71.59 ± 3.11	−50.02 ± 2.98
EEL	−20.98 ± 6.09	−12.45 ± 3.17	−86.16 ± 22.28	−7.04 ± 7.19	−26.76 ± 5.50	−23.69 ± 6.75
EPB	49.05 ± 6.39	49.13 ± 3.55	105.66 ± 23.35	46.74 ± 8.46	58.12 ± 5.08	38.00 ± 6.10
ENPOLAR	−50.90 ± 1.81	−55.88 ± 1.18	−34.60 ± 2.83	−55.08 ± 2.34	−50.88 ± 1.38	−39.96 ± 1.50
EDISPER	86.67 ± 2.01	91.08 ± 1.34	58.67 ± 4.56	93.79 ± 2.76	87.51 ± 1.86	65.74 ± 1.74
Δ*G*_gas_	−89.42 ± 7.00	−85.26 ± 4.33	−126.20 ± 24.77	−81.81 ± 8.72	−98.35 ± 6.62	−73.71 ± 7.17
Δ*G*_solv_	84.81 ± 6.57	84.33 ± 3.85	129.73 ± 24.70	85.45 ± 9.16	94.76 ± 5.64	63.79 ± 6.42
ΔTotal	−4.61 ± 5.73	−0.92 ± 4.43	3.53 ± 4.74	3.65 ± 5.43	−3.59 ± 4.94	−9.93 ± 4.79

Subsequent MM‐PBSA analysis of the Y537S mutant ERα complexes revealed significant alterations in ligand interactions, as detailed in Table 3. The compounds ibs‐18821 and ibs‐04156 exhibited the most favorable binding energies among the candidates, with ΔTotal values of −9.45 ± 6.56 and −9.17 ± 5.52 kcal/mol, respectively. A strong binding potential was also observed for ibs‐37312, which had a ΔTotal of −6.85 ± 5.53 kcal/mol. In comparison, the mutant‐active reference compound lasofoxifene displayed a highly favorable binding free energy of −12.24 ± 4.96 kcal/mol.

**TABLE 3:** Binding free energy components (kcal/mol) for Y537S mutant ERα–ligand complexes, as determined by MM‐PBSA calculations.

Component	ibs‐04156	ibs‐07025	ibs‐18821	ibs‐19591	ibs‐37312	Lasofoxifene
VDW	−76.71 ± 5.38	−70.61 ± 3.33	−93.04 ± 4.44	−96.29 ± 6.17	−70.21 ± 3.79	−64.82 ± 3.02
EEL	−18.84 ± 5.58	−6.72 ± 3.91	−48.52 ± 7.70	−36.52 ± 10.53	−8.68 ± 4.44	−14.53 ± 5.05
EPB	48.97 ± 4.78	41.32 ± 5.05	88.95 ± 6.17	85.28 ± 15.05	37.71 ± 4.15	37.39 ± 3.30
ENPOLAR	−55.81 ± 2.32	−52.25 ± 1.78	−63.68 ± 1.62	−67.52 ± 2.78	−49.63 ± 1.82	−44.38 ± 0.71
EDISPER	93.22 ± 3.25	87.79 ± 2.18	106.84 ± 2.09	112.21 ± 3.22	83.96 ± 1.99	74.11 ± 1.10
Δ*G*_gas_	−95.55 ± 5.85	−77.33 ± 5.22	−141.56 ± 8.87	−132.81 ± 13.51	−78.90 ± 5.83	−79.36 ± 4.41
Δ*G*_solv_	86.38 ± 5.32	76.85 ± 5.52	132.11 ± 6.63	129.97 ± 15.45	72.05 ± 4.42	67.11 ± 3.88
ΔTotal	−9.17 ± 5.52	−0.48 ± 5.23	−9.45 ± 6.56	−2.84 ± 9.30	−6.85 ± 5.53	−12.24 ± 4.96

**FIGURE 9:**
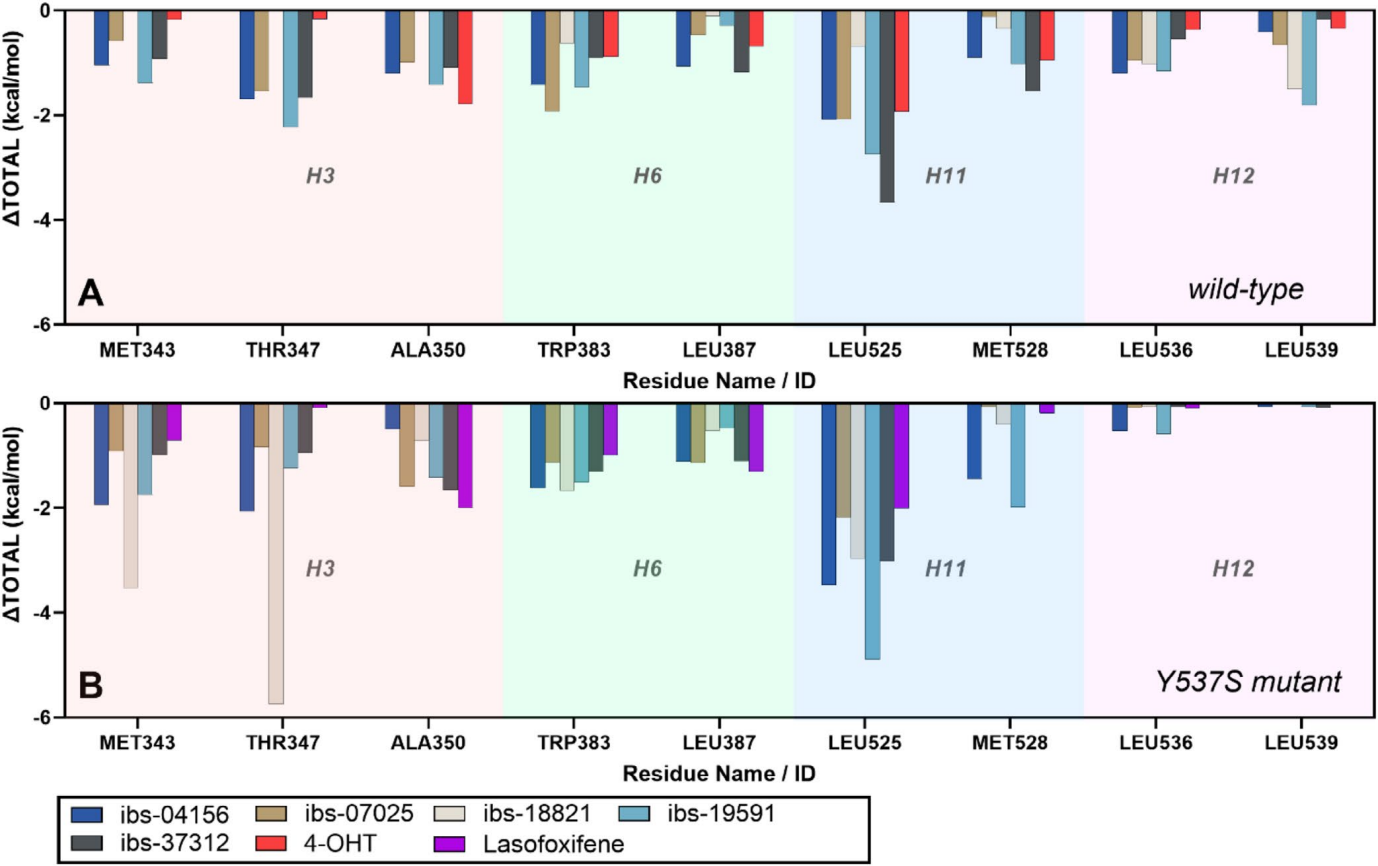
Comparative analysis of per‐residue binding free energy contributions for key residues within the ERα active site. The plots illustrate the energetic profiles for the phytochemical candidates and clinical controls in complex with (A) wild‐type ERα and (B) the Y537S mutant. Highlighted sections correspond to residues located within helices H3, H6, H11, and H12.

To elucidate the impact of the Y537S mutation on ligand binding, the total binding free energy was decomposed into contributions from individual key residues using MM‐PBSA calculations. This analysis reveals the specific residues responsible for the altered binding affinities in the mutant ERα, as illustrated in Figure 9.

The results indicate a significant redistribution of interaction energies upon mutation. The H3 alpha‐helix region emerged as a primary locus for enhanced binding, particularly for the ligand ibs‐18821. The energetic contributions from residues Met343, Thr347, and Ala350 to the binding of ibs‐18821 were strengthened by approximately 3.55, 5.73, and 0.77 kcal/mol, respectively, in the mutant complex. Further stabilization for ibs‐18821 was observed in the H11 region, with contributions from Leu525 and Met528 increasing by 2.15 and 0.96 kcal/mol. The ligand ibs‐04156 also benefited from enhanced interactions with these same H11 residues, with energy contributions improving by 1.38 and 0.56 kcal/mol.

Conversely, the H12 region, which contains the Y537S mutation site, exhibited destabilizing effects on the binding of all tested ligands. Interactions with residues Leu536 and Leu539 became less favorable for the entire set of compounds, including the reference 4‐OHT. The binding of ibs‐18821 was notably hindered in this region, with its interaction energies decreasing by 0.57 kcal/mol at Leu536 and 1.75 kcal/mol at Leu539 compared to the wild‐type. This finding suggests that while the Y537S mutation can indirectly enhance ligand interactions in distal helices, it directly introduces unfavorable interactions within the H12 region for both the novel ibs‐series compounds and the established antagonist lasofoxifene.

#### 3.3.4 Lead Selection and Evaluation

To systematically rank the candidate compounds, a weighted scoring function was developed based on seven key metrics derived from post‐simulation analysis. As detailed in Table S1–S4, these metrics include the RMSD, RMSF, *R*_g_, SASA, number of hydrogen bonds, number of interacting residue pairs, and the MM‐PBSA binding free energy.

**FIGURE 10:**
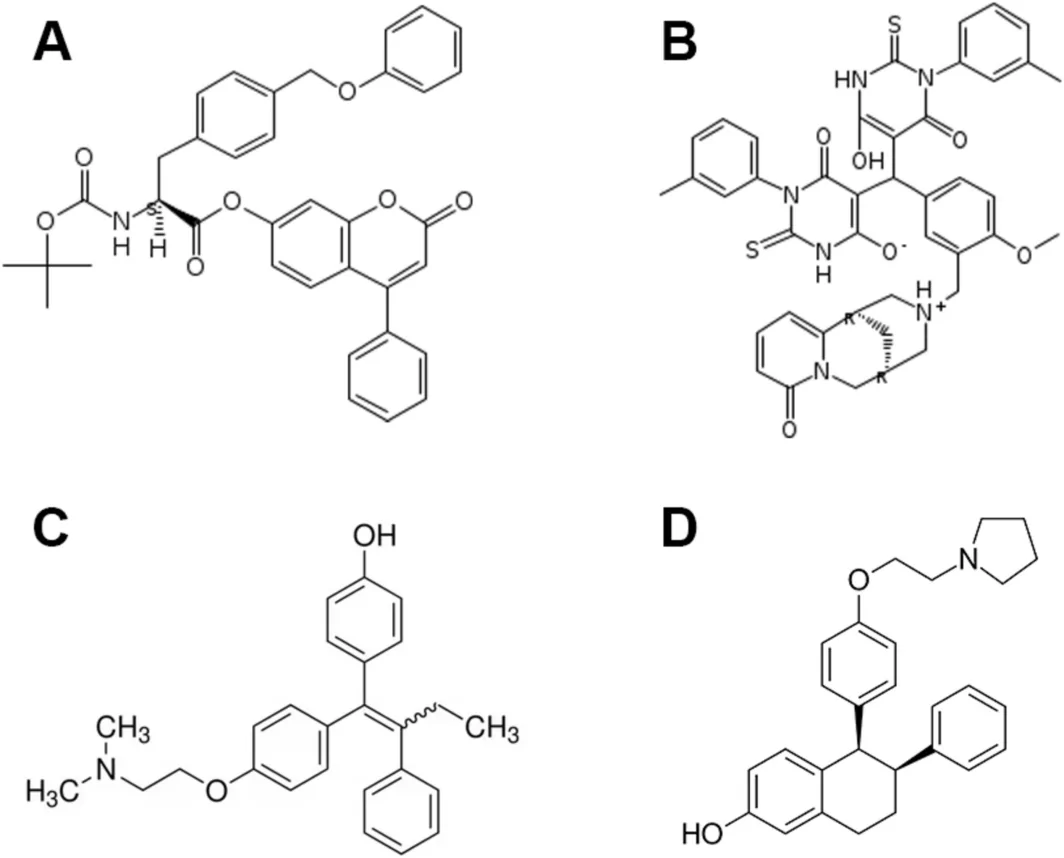
Chemical structures of the identified lead compounds and the reference controls. (A) ibs‐04156, (B) ibs‐18821, (C) 4‐hydroxytamoxifen, and (D) lasofoxifene.

Recognizing the differential impact of these parameters on inhibitor potential, a distinct weighting factor was assigned to each. Metrics of moderate influence—RMSD, RMSF, hydrogen bonds, and interacting residues—were each assigned a weight of 0.1. Parameters with a lesser impact, *R*_g_ and SASA, were given a weight of 0.05. The MM‐PBSA binding free energy, considered the primary determinant of binding affinity, received the highest weight of 0.5. The final score for each ligand was calculated as the weighted sum of its ranks across all seven metrics. A lower score indicates a more favorable profile. Based on this evaluation, ibs‐18821 and ibs‐04156 were identified as two strong potent inhibitors for the Y537S mutant ERα, achieving final scores of 1.75 and 2.95, respectively. The 2D chemical structures of the two top‐ranked candidates, ibs‐04156 and ibs‐18821, along with the reference controls 4‐OHT and lasofoxifene, are depicted in Figure 10.

**FIGURE 11:**
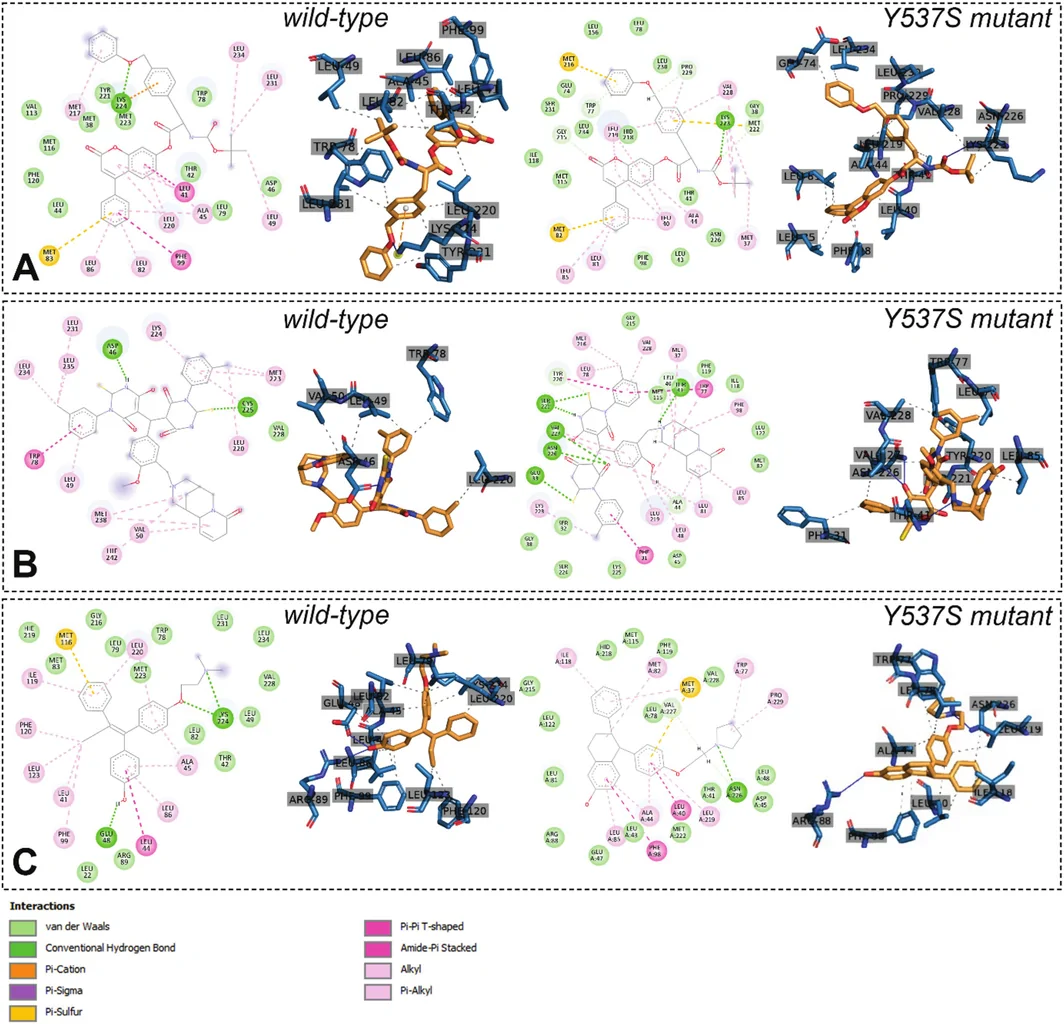
2D and 3D representations of molecular interaction profiles within ERα–ligand complexes. Panel (A) depicts interactions for ibs‐04156 and (B) for ibs‐18821. For these candidates, interactions with both wild‐type ERα (left) and the Y537S mutant ERα (right) are presented. Panel (C) depicts the clinical controls: 4‐OHT with the wild‐type ERα (left) and lasofoxifene with the Y537S mutant ERα (right). The legend indicates different types of non‐covalent interactions.

Analysis of the protein–ligand interaction patterns revealed a corresponding change in the number of residues engaging with the ligands upon mutation. As shown in Figure 11, the number of residues interacting with ibs‐04156 increased from 12 in the wild‐type complex to 14 in the mutant. For ibs‐18821, a more substantial increase from 5 to 10 interacting residues was observed. In contrast, the clinical benchmarks demonstrated distinct baseline interaction networks, with 4‐OHT interacting with 12 residues in the wild‐type system and lasofoxifene interacting with 10 residues in the mutant receptor.

#### 3.3.5 Principal Component Analysis

**FIGURE 12:**
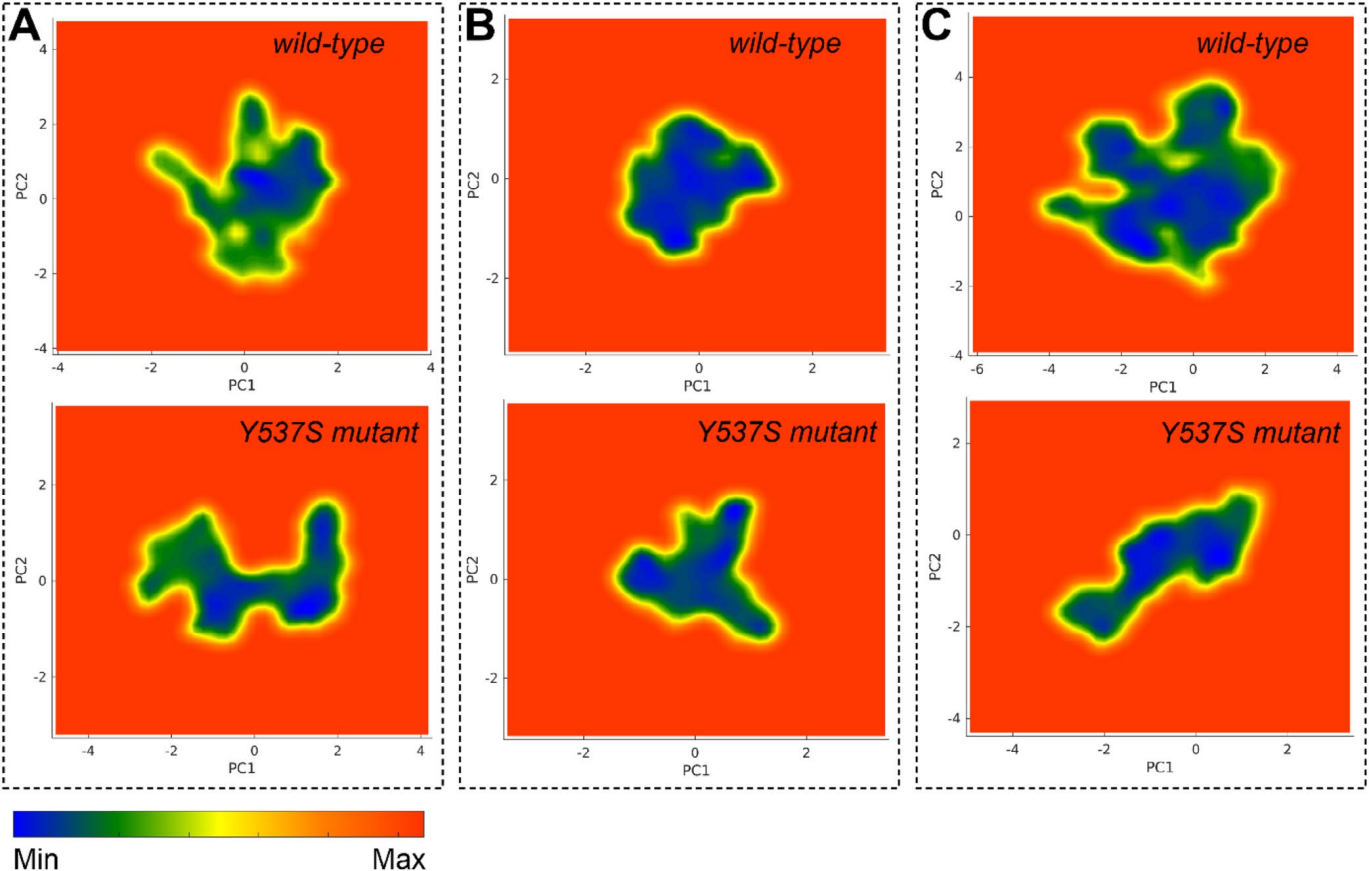
Principal component analysis of the binding free energy landscapes for wild‐type and Y537S mutant ERα complexed with various ligands. The respective ligands are: (A) ibs‐04156, (B) ibs‐18821, and (C) 4‐OHT (wild‐type) and lasofoxifene (mutant).

Principal component analysis was utilized to elucidate the distribution of binding free energies along the two major principal components, PC1 and PC2. The analysis revealed distinct alterations in the conformational landscapes of the studied compounds upon mutation. As illustrated in Figure 12, for ibs‐04156, the low‐energy region expanded from two primary basins in the wild‐type to three basins in the Y537S mutant, suggesting an increase in the number of potent binding conformations. This was accompanied by a decrease in the minimum binding free energy from −22.70 kcal/mol in the wild‐type to −26.08 kcal/mol in the mutant. In the case of ibs‐18821, a large, aggregated low‐energy basin in the wild‐type system transformed into multiple, dispersed basins in the mutant. A significant reduction in the minimum binding free energy was observed, from −12.92 to −31.89 kcal/mol, which indicates the formation of a highly stable binding pose. Conversely, the clinical controls established distinct baseline landscapes for each system. The reference compound 4‐hydroxytamoxifen exhibited multiple dispersed low‐energy basins in the wild‐type system with a minimum binding energy of −26.13 kcal/mol. In the Y537S mutant, the mutant‐active control lasofoxifene displayed two primary basins.

#### 3.3.6 Conformational Analysis of Helix 12

**FIGURE 13:**
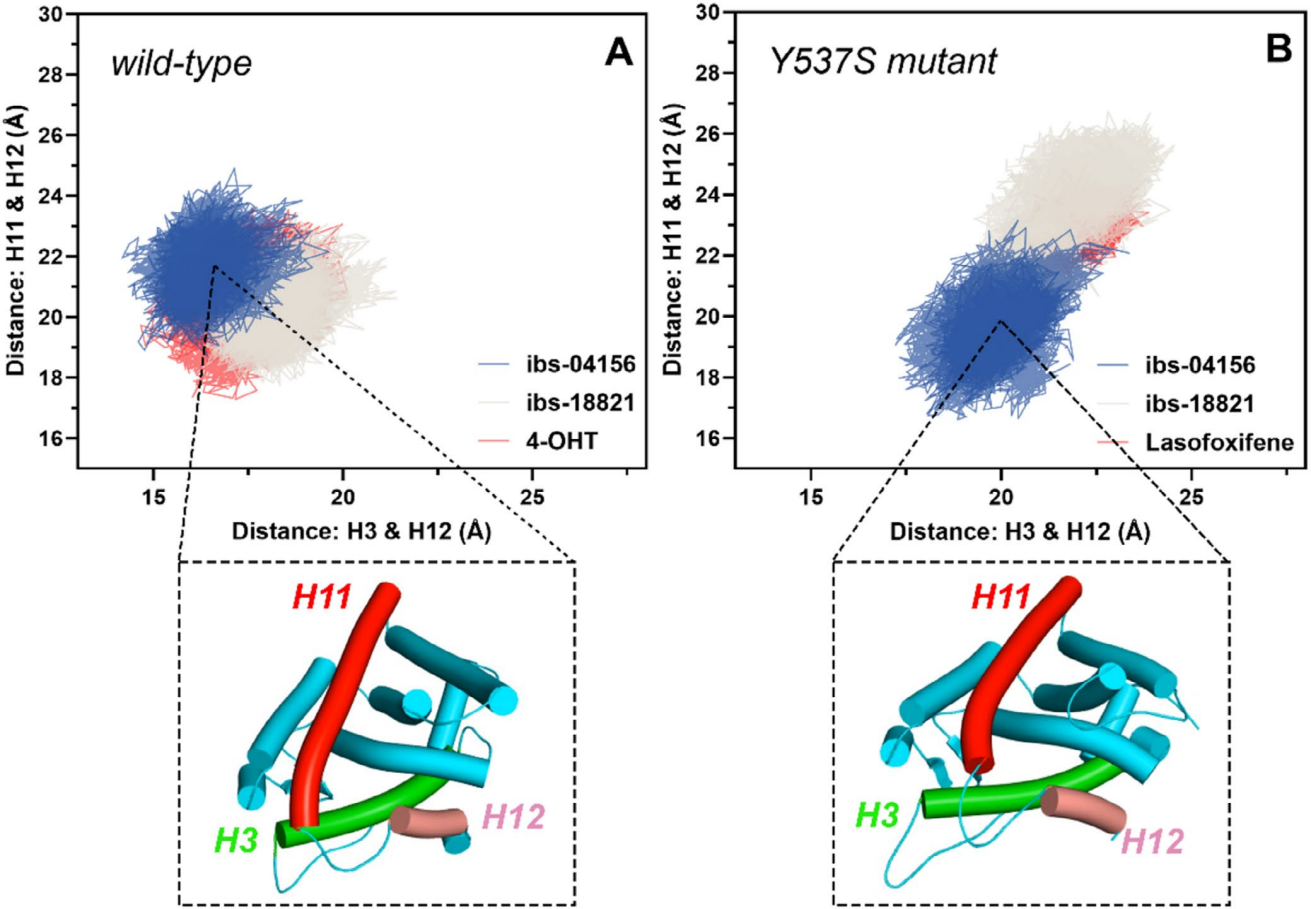
Conformational landscape of ERα based on helical distances. The plots illustrate the relationship between the center‐of‐mass distances of the H3–H12 interface (*x*‐axis) and the H11–H12 interface (*y*‐axis). The analysis compares the conformational space of (A) wild‐type ERα and (B) the Y537S mutant when complexed with ibs‐04156, ibs‐18821, and the clinical controls (4‐OHT for the wild‐type and lasofoxifene for the mutant).

To evaluate the structural mechanism by which ibs‐18821 overcomes the Y537S mutation, the conformational dynamics of Helix 12 were monitored relative to Helix 3 and Helix 11. Center‐of‐mass distances between H12 and these helices were measured to map ligand‐induced displacement in both wild‐type and mutant systems, as illustrated in Figure 13. In the wild‐type ERα simulations, ibs‐18821 maintained an H11–H12 distance of approximately 20.2 Å. This value is comparable to the 20.7 Å observed for the reference antagonist 4‐OHT. These data suggest that within the non‐mutated receptor, ibs‐18821 stabilizes a conformation spatially analogous to that of standard endocrine therapies.

Conversely, a distinct divergence was observed in the Y537S mutant system. Ibs‐04156 failed to induce significant separation, evidenced by an H11–H12 distance of 19.6 Å. Lasofoxifene exhibited a moderate increase and ibs‐18821 induced a marked displacement of H12. The H11–H12 distance for the ibs‐18821–mutant complex increased significantly to an average of 23.3 Å. This substantial spatial deviation indicates that ibs‐18821 exerts a distinct steric influence on the mutant receptor and effectively disrupts the H11–H12 interface to a greater extent. By inducing this extended conformation of H12, ibs‐18821 likely prevents stabilization of the agonist‐like state favored by the Y537S mutation, thereby restoring inhibitory efficacy in a context where conventional agents exhibit limited activity.

## 4 Discussion

Phytomedicines are an established therapeutic option in oncology (Izzo and Stefanska 2025). The clinical importance of these products has been proven by compounds like paclitaxel and the vinca alkaloids (Banyal et al. 2023; Cragg and Pezzuto 2016). Naturally derived compounds hold a vast structural diversity (Atanasov et al. 2021). These libraries are composed of millions of unique chemical scaffolds, which are different from traditional synthetic compound collections (Tay et al. 2023). Combining high‐throughput in silico screening with large natural libraries gathers the strengths of both methods, which allows for the quick identification (Ayon 2023). The Y537S mutant of the estrogen receptor alpha is one such target, conferring resistance to standard endocrine therapies (Fiorillo et al. 2018). The estrogen receptor is a frequent target for natural compound studies, which led to the identification of the soybean isoflavone metabolite equol, resveratrol, and genistein. These compounds are known to modulate the activity of ERα and ERβ (Bak et al. 2016; Kunnumakkara et al. 2008; Lecomte et al. 2017; Muthyala et al. 2004; Nwachukwu et al. 2014).

ESR1 gene mutations are a major cause of acquired resistance to endocrine therapy in metastatic breast cancer (Ahn et al. 2022). The Y537S mutation forms a new hydrogen bond in the Helix 12 region (Fanning et al. 2016). This H‐bond stabilizes ERα in a constantly active, agonist‐like conformation without the estrogen (Fanning et al. 2016). This stabilization leads to uncontrolled and ligand‐independent cancer cell proliferation (Fanning et al. 2016). It also causes a conformational shift that cancels the inhibitory effect of selective estrogen receptor modulators like tamoxifen (Fiorillo et al. 2018). A new compound with very high binding affinity for the ligand‐binding pocket of the Y537S ERα mutant is needed to solve this problem. The structural diversity of ethnomedicinal resources offers a good pool of potential candidates. In silico methods permit the quantitative and systematic evaluation of many of these compounds. There is no natural product that is currently validated as an effective inhibitor of mutated ERα, highlighting a major gap in the research.

The molecular dynamics of the Y537S ERα mutant were analyzed in this study. Two natural compounds, ibs‐18821 and ibs‐04156, were flagged as potential inhibitors because of their good binding characteristics. Post‐molecular dynamics analysis showed these compounds have a strong binding potential to the drug‐resistant mutant ERα. Notably, ibs‐04156 distinguished itself as the most promising overall candidate due to its remarkable consistency across both the wild‐type and mutated systems. As detailed in the composite scoring (Tables S2 and S4), ibs‐04156 maintained a top‐tier ranking in both receptor states. Its structural adaptability is evidenced by the principal component analysis (Figure 12), where its low‐energy binding basins expanded upon mutation, alongside a favorable decrease in minimum binding free energy. This adaptability is supported by a robust interaction network; the number of residues interacting with ibs‐04156 increased from 12 in the wild‐type to 14 in the mutant complex (Figure 11), driving a highly favorable MM‐PBSA binding free energy of −9.17 kcal/mol in the mutated state (Table 3). This reliable performance establishes ibs‐04156 as a potent dual‐action inhibitor capable of targeting both states of the receptor.

Conversely, ibs‐18821 emerged as a potent candidate specifically targeting the mutated system through a distinct structural mechanism. It exhibits a superior binding affinity driven largely by allosteric engagement with distal helices. Figure 6 shows that ibs‐18821 causes a significant compaction of the mutated ERα structure. The resulting protein is highly compact, comparable to the conformation induced by the mutant‐active clinical control, lasofoxifene. The mechanism is shown in Figure 8, which details the formation of many new nonpolar contacts between the Y537S binding pocket residues and the atoms of ibs‐18821. These contacts directly contribute to the better binding free energy of ibs‐18821, as detailed in Table 3. The analysis in Figure 9 suggests this stabilizing effect is not from direct interactions in the mutated H12 region. It comes from allosteric interactions between the ligand and distant helices, mainly Helix 3 and Helix 11. As shown in Figure 13, the center‐of‐mass distance between helices H11 and H12 exhibited a critical deviation. The complex of ibs‐18821 with the Y537S ERα mutant showed the largest separation between these two helices. Principal component analysis supports these results. While the clinical control lasofoxifene establishes a distinct baseline with two primary basins in the mutant system, the ibs‐18821 complex engages multiple dispersed low‐energy states, indicating a highly stable, albeit conformationally distinct, binding profile. Furthermore, analyzing these MM‐PBSA energetic profiles alongside conformational alterations aligns with recent computational investigations of mutated ERα, which similarly emphasize the importance of binding free energy distribution in understanding drug resistance mechanisms (Bouricha and Hakmi 2024).

This study has faced certain limitations. The in vitro cytotoxicity experiments were performed exclusively using the MCF‐7 cell line, which expresses wild‐type ERα. Consequently, the inhibitory activity of these compounds against the Y537S mutant receptor remains a computational prediction and requires direct experimental validation. In silico modeling approximates the interactions and kinetics between ERα mutants and their phytochemical ligands. The MM‐PBSA method is challenging to generate results that match experimental binding affinities (Genheden and Ryde 2015). Physiological conditions also add variables that affect these interactions (Adediwura et al. 2024). ERα is a protein with many functions (Zhou et al. 2012). Mutation impacts not only protein stability, but also processes like homodimer formation and DNA binding (Harrod et al. 2017). The influence of the full‐length protein of a mutated ERα demands complex validation (Gates et al. 2018). These areas require further investigation in future studies.

## 5 Conclusion

This study identified potential naturally derived inhibitors for the Y537S mutant of ERα through a combination of high‐throughput virtual screening and molecular dynamics simulations. Among the candidates, ibs‐04156 emerged as the most promising overall compound, demonstrating consistent stability and highly favorable binding affinities across both the wild‐type and mutated receptors. Post‐MD analysis revealed that ibs‐04156 maintains a robust interaction network in both systems, establishing it as a reliable dual‐action inhibitor. Its potential to overcome endocrine therapy resistance conferred by the Y537S mutation makes it a strong candidate for further experimental validation as a novel breast cancer therapeutic.

## Author Contributions

**Dejun Jiang:** methodology, software, data curation, formal analysis, writing – original draft, visualization. **Oh Wook Kwon:** methodology, investigation, validation, data curation. **Hanbin Joe:** methodology, resources. **Euijeong Shin:** methodology, investigation. **Sungjoon Cho:** conceptualization, visualization. **Hyuk‐Ku Kwon:** writing – review and editing, supervision. **Youngjin Choi:** conceptualization, data curation, supervision, validation, writing – original draft, writing – review and editing, project administration, funding acquisition.

## Funding

This research was supported by the Basic Science Research Program through the National Research Foundation of Korea (NRF), funded by the Ministry of Education (RS‐2021‐NR066286).

## Conflicts of Interest

The authors declare no conflicts of interest.

## Supporting information


**Figure S1:** Distribution of docking affinities for 15,431 compounds screened against Y537S‐mutated estrogen receptor α using AutoDock4. The dashed red line indicates the selection threshold of −12 kcal/mol, representing the criterion for evaluating compounds.
**Table S1:** Molecular dynamics stability and interaction metrics of the wild‐type ERα complexed with the leading IBS compound compared to the reference chemical, 4‐OHT.
**Table S2:** Weighted composite scoring of the post‐MD analysis and overall ranking of the leading candidates and 4‐OHT complexed with the wild‐type ERα.
**Table S3:** Molecular dynamics stability and interaction metrics of the Y537S‐mutated ERα complexed with the leading IBS compound compared to the reference chemical, lasofoxifene.
**Table S4:** Weighted composite scoring of the post‐MD analysis and overall ranking of the leading candidates and lasofoxifene complexed with the Y537S‐mutated ERα.

## Data Availability

Data are contained within the article.
