# Supplementary material for: Discovery of ERα‐Targeting Phytochemicals With In Vitro Cytotoxicity and Computational Prediction of Y537S Mutant Inhibition
Source: Chem Biol Drug Des. 2026 Sep 3;108(3):e70393. doi: 10.1111/cbdd.70393 (PMC13542627; doi:10.1111/cbdd.70393)
Supplement: Supplementary file 1 [file CBDD-108-e70393-s001.docx]

**Figure S1.** Distribution of docking affinities for 15,431 compounds screened against Y537S-mutated estrogen receptor α using AutoDock4. The dashed red line indicates the selection threshold of -12 kcal/mol, representing the criterion for evaluating compounds.

**Table S1.** Molecular dynamics stability and interaction metrics of the wild-type ERα complexed with the leading IBS compound compared to the reference chemical, 4-OHT.

| **Compound** | **RMSD (Å)** | | **RMSF (Å)** | | **R_g_ (Å)** | | **SASA (Å^2^)** | | **H-bonds** | | **Contacts** | | **ΔG (kcal/mol)** | |
| --- | --- | --- | --- | --- | --- | --- | --- | --- | --- | --- | --- | --- | --- | --- |
|  | **Mean ± SD** | **Rank** | **Mean ± SD** | **Rank** | **Mean ± SD** | **Rank** | **Mean ± SD** | **Rank** | **Mean ± SD** | **Rank** | **Mean ± SD** | **Rank** | **Mean ± SD** | **Rank** |
| ibs-04156 | 2.02 ± 0.13 | 3 | 1.26 ± 0.98 | 5 | 18.40 ± 0.11 | 2 | 1866.38 ± 92.38 | 5 | 0.44 ± 0.72 | 6 | 213.39 ± 34.75 | 4 | -4.62 ± 5.73 | 2 |
| ibs-07025 | 2.62 ± 0.12 | 5 | 1.10 ± 0.84 | 1 | 18.35 ± 0.07 | 1 | 1877.29 ± 66.90 | 6 | 1.03 ± 0.98 | 4 | 164.06 ± 24.95 | 5 | -0.96 ± 4.44 | 4 |
| ibs-18821 | 2.95 ± 0.19 | 6 | 1.15 ± 0.96 | 4 | 18.58 ± 0.07 | 4 | 1675.73 ± 73.15 | 3 | 2.14 ± 0.94 | 1 | 105.67 ± 25.07 | 6 | 3.50 ± 4.74 | 5 |
| ibs-19591 | 2.02 ± 0.22 | 2 | 1.34 ± 1.44 | 6 | 18.48 ± 0.08 | 3 | 1511.67 ± 76.92 | 2 | 0.71 ± 0.94 | 5 | 267.47 ± 39.28 | 3 | 3.65 ± 5.43 | 6 |
| ibs-37312 | 1.83 ± 0.14 | 1 | 1.12 ± 1.20 | 2 | 18.67 ± 0.07 | 5 | 1831.84 ± 81.74 | 4 | 1.46 ± 1.11 | 3 | 291.44 ± 63.31 | 1 | -3.56 ± 4.95 | 3 |
| 4-OHT | 2.46 ± 0.17 | 4 | 1.14 ± 1.06 | 3 | 18.70 ± 0.11 | 6 | 1497.28 ± 69.57 | 1 | 1.75 ± 0.94 | 2 | 290.37 ± 24.26 | 2 | -9.91 ± 4.79 | 1 |

**Table S2.** Weighted composite scoring of the post-MD analysis and overall ranking of the leading candidates and 4-OHT complexed with the wild-type ERα.

| **Compound** | **RMSD** | | **RMSF** | | **R_g_** | | **SASA** | | **H-bonds** | | **Contacts** | | **ΔG** | | **Overall Score** | **Overall Rank** |
| --- | --- | --- | --- | --- | --- | --- | --- | --- | --- | --- | --- | --- | --- | --- | --- | --- |
|  | **Rank** | **Weighted (w=0.10)** | **Rank** | **Weighted (w=0.10)** | **Rank** | **Weighted (w=0.05)** | **Rank** | **Weighted (w=0.05)** | **Rank** | **Weighted (w=0.10)** | **Rank** | **Weighted (w=0.10)** | **Rank** | **Weighted (w=0.50)** |  |  |
| 4-OHT | 4 | 0.40 | 3 | 0.30 | 6 | 0.30 | 1 | 0.05 | 2 | 0.20 | 2 | 0.20 | 1 | 0.50 | 1.95 | 1 |
| ibs-37312 | 1 | 0.10 | 2 | 0.20 | 5 | 0.25 | 4 | 0.20 | 1 | 0.10 | 3 | 0.30 | 3 | 1.50 | 2.65 | 2 |
| ibs-04156 | 3 | 0.30 | 5 | 0.50 | 2 | 0.10 | 5 | 0.25 | 4 | 0.40 | 6 | 0.60 | 2 | 1.00 | 3.15 | 3 |
| ibs-07025 | 5 | 0.50 | 1 | 0.10 | 1 | 0.05 | 6 | 0.30 | 5 | 0.50 | 4 | 0.40 | 4 | 2.00 | 3.85 | 4 |
| ibs-18821 | 6 | 0.60 | 4 | 0.40 | 4 | 0.20 | 3 | 0.15 | 6 | 0.60 | 1 | 0.10 | 5 | 2.50 | 4.55 | 5 |
| ibs-19591 | 2 | 0.20 | 6 | 0.60 | 3 | 0.15 | 2 | 0.10 | 3 | 0.30 | 5 | 0.50 | 6 | 3.00 | 4.85 | 6 |

**Table S3.** Molecular dynamics stability and interaction metrics of the Y537S-mutated ERα complexed with the leading IBS compound compared to the reference chemical, lasofoxifene.

| **Compound** | **RMSD (Å)** | | **RMSF (Å)** | | **R_g_ (Å)** | | **SASA (Å^2^)** | | **H-bonds** | | **Contacts** | | **ΔG (kcal/mol)** | |
| --- | --- | --- | --- | --- | --- | --- | --- | --- | --- | --- | --- | --- | --- | --- |
|  | **Mean ± SD** | **Rank** | **Mean ± SD** | **Rank** | **Mean ± SD** | **Rank** | **Mean ± SD** | **Rank** | **Mean ± SD** | **Rank** | **Mean ± SD** | **Rank** | **Mean ± SD** | **Rank** |
| ibs-04156 | 2.95 ± 0.20 | 4 | 1.17 ± 0.90 | 5 | 18.14 ± 0.10 | 5 | 2550.28 ± 83.99 | 4 | 1.13 ± 0.96 | 3 | 293.00 ± 35.55 | 4 | -9.17 ± 5.52 | 3 |
| ibs-07025 | 2.52 ± 0.18 | 2 | 1.11 ± 0.93 | 3 | 18.05 ± 0.13 | 4 | 1941.14 ± 90.81 | 2 | 0.49 ± 0.77 | 6 | 259.76 ± 36.42 | 5 | -0.48 ± 5.23 | 5 |
| ibs-18821 | 3.18 ± 0.19 | 5 | 1.05 ± 0.63 | 1 | 17.88 ± 0.07 | 2 | 2604.35 ± 79.13 | 5 | 2.15 ± 1.26 | 1 | 514.37 ± 46.95 | 1 | -9.45 ± 6.56 | 2 |
| ibs-19591 | 2.76 ± 0.21 | 6 | 1.36 ± 1.52 | 4 | 18.36 ± 0.08 | 6 | 2465.22 ± 100.44 | 6 | 1.95 ± 1.03 | 4 | 430.62 ± 57.87 | 3 | -2.84 ± 9.30 | 6 |
| ibs-37312 | 2.50 ± 0.11 | 1 | 1.28 ± 1.15 | 6 | 18.01 ± 0.11 | 3 | 1976.14 ± 104.78 | 3 | 0.99 ± 0.92 | 5 | 252.43 ± 32.28 | 6 | -6.85 ± 5.53 | 4 |
| Lasofoxifene | 2.90 ± 0.10 | 3 | 1.07 ± 0.73 | 2 | 17.84 ± 0.10 | 1 | 1463.10 ± 75.20 | 1 | 1.61 ± 1.08 | 2 | 447.46 ± 29.65 | 2 | -12.24 ± 4.96 | 1 |

**Table S4.** Weighted composite scoring of the post-MD analysis and overall ranking of the leading candidates and lasofoxifene complexed with the Y537S-mutated ERα.

| **Compound** | **RMSD** | | **RMSF** | | **R_g_** | | **SASA** | | **H-bonds** | | **Contacts** | | **ΔG** | | **Overall Score** | **Overall Rank** |
| --- | --- | --- | --- | --- | --- | --- | --- | --- | --- | --- | --- | --- | --- | --- | --- | --- |
|  | **Rank** | **Weighted (w=0.10)** | **Rank** | **Weighted (w=0.10)** | **Rank** | **Weighted (w=0.05)** | **Rank** | **Weighted (w=0.05)** | **Rank** | **Weighted (w=0.10)** | **Rank** | **Weighted (w=0.10)** | **Rank** | **Weighted (w=0.50)** |  |  |
| Lasofoxifene | 3 | 0.30 | 2 | 0.10 | 1 | 0.05 | 1 | 0.05 | 2 | 0.20 | 2 | 0.20 | 1 | 0.50 | 1.50 | 1 |
| ibs-18821 | 5 | 0.50 | 1 | 0.30 | 2 | 0.10 | 5 | 0.25 | 1 | 0.10 | 1 | 0.10 | 2 | 1.00 | 2.15 | 2 |
| ibs-04156 | 4 | 0.40 | 5 | 0.50 | 5 | 0.10 | 4 | 0.20 | 3 | 0.30 | 4 | 0.40 | 3 | 1.50 | 3.55 | 3 |
| ibs-37312 | 1 | 0.10 | 6 | 0.40 | 3 | 0.25 | 3 | 0.15 | 5 | 0.50 | 6 | 0.60 | 4 | 2.00 | 4.10 | 4 |
| ibs-07025 | 2 | 0.20 | 3 | 0.60 | 4 | 0.30 | 2 | 0.10 | 6 | 0.60 | 5 | 0.50 | 5 | 2.50 | 4.40 | 5 |
| ibs-19591 | 6 | 0.60 | 4 | 0.20 | 6 | 0.15 | 6 | 0.30 | 4 | 0.40 | 3 | 0.30 | 6 | 3.00 | 5.30 | 6 |
